# Draft Genome Sequences of Pseudomonas Isolates Derived from Glyphosate-Treated Sediments

**DOI:** 10.1128/mra.01147-22

**Published:** 2023-04-26

**Authors:** Sabryna C. Cadavillo, Morgan P. Stroud, Madison Newman, Jovan Zigic, Samuel D. Roman, Bernadette J. Connors

**Affiliations:** a Science Department, Dominican University New York, Orangeburg, New York, USA; b Edgemont High School, Scarsdale, New York, USA; University of Delaware College of Engineering

## Abstract

Reported here are the genome sequences of Pseudomonas isolates that were derived from glyphosate-treated sediment microcosms. Genomes were assembled using workflows available through the Bacterial and Viral Bioinformatics Resource Center (BV-BRC). The genomes of eight Pseudomonas isolates were sequenced, with genomes ranging from 5.9 to 6.3 Mb.

## ANNOUNCEMENT

**FIG 1:**
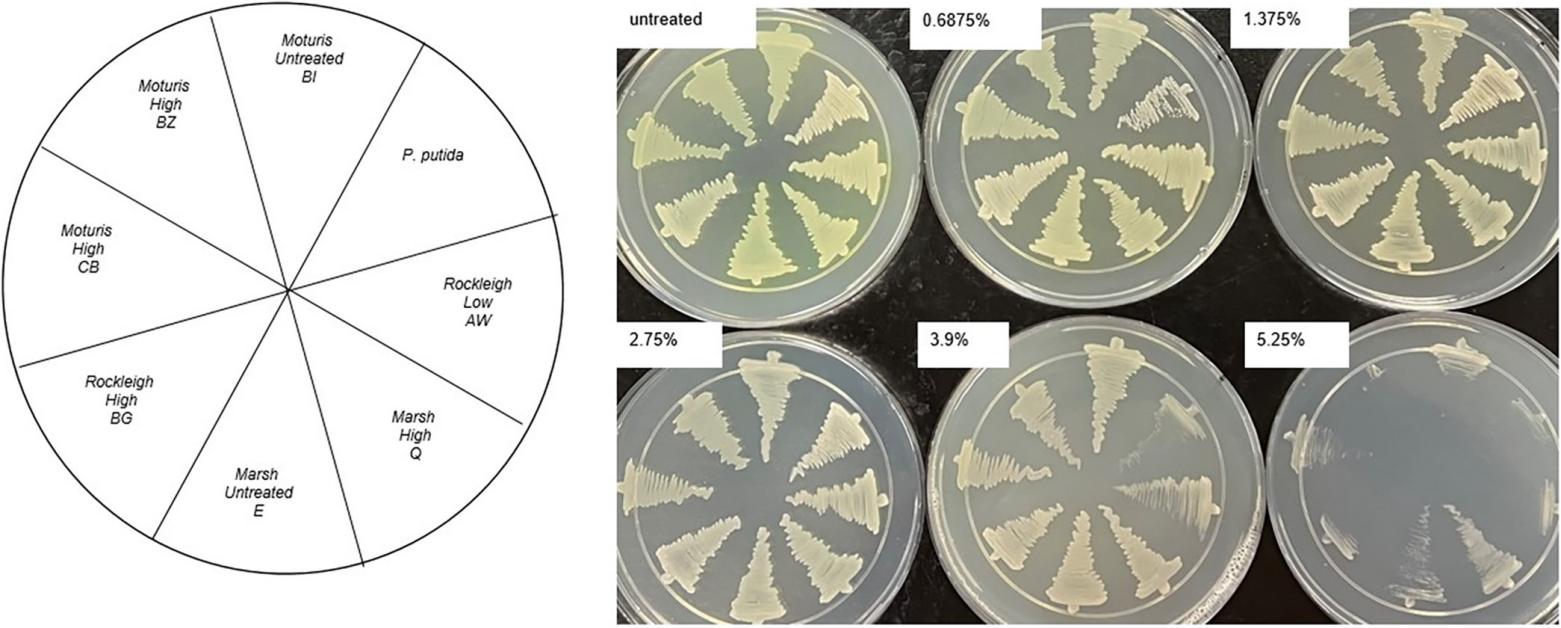
Growth of isolates in glyphosate. Pseudomonas isolates were streaked on Mueller-Hinton agar containing various concentrations of glyphosate (0.675% to 5.25% [vol/vol]). Growth on these media was used to determine the concentrations of glyphosate used in subsequent experiments.

Glyphosate is a widely used broad-spectrum organophosphorus herbicide (1). Several studies have shown that Pseudomonas communities are enhanced in glyphosate-contaminated waters and soils (2–4), with some research demonstrating adverse effects on the phenotypic characteristics of these bacteria (5, 6). In the current study, glyphosate microcosms were created with 100 mL of sediment slurry derived from a suburban creek. Sediment was collected in sterile containers by submerging the closed containers under the surface of the substrate. Microcosms were created in sterile glass containers that were loosely capped. The sediments were kept at room temperature and left untreated or subjected to the addition of commercial-grade glyphosate at concentrations of 0.675% (low), 3.125% (medium), or 5.25% (high) (vol/vol) for 1 week. No other amendments were included. A 1:10 serial dilution of the slurry was then created in sterile water using ~0.5 g as the starting material. Aliquots of each dilution were spread on eosin methylene blue (EMB) medium (BD BBL EMB agar [Levine]; Fisher Scientific) and incubated for 24 h at 32°C. Isolates chosen for full genome sequencing were selected following 16S rRNA gene analysis using universal primer sets that recognize the V1 to V9 regions (Azenta Life Sciences). An NCBI BLASTn search (7) determined that the isolates chosen for further analysis belong to the genus Pseudomonas (see Table 1 for the GenBank reference accession numbers). These isolates, along with a commercial Pseudomonas putida control strain purchased from Carolina Biologicals, were inoculated onto Mueller-Hinton agar containing commercial glyphosate to verify their ability to grow in the presence of the herbicide (Fig. 1).

**TABLE 1:** Basic statistical assembly and annotation analyses

Isolate	Collection location coordinate	SRA accession no.	GenBank nucleotide accession no.	Assembly size (bp)	No. of contigs	GC content (%)	No. of reads	No. of identified genes	No. of coding sequences	No. of predicted tRNAs	GenBank reference accession no.	Species (GenBank accession no.) of top hit
Rockleigh low (isolate AW)	41.007620, −73.940000	SRR22223272	JAPFCE000000000	6,333,037	229	62.26	2,020,594	5,661	5,567	67	MF111944.1	Unclassified Pseudomonas (CP087181.1)
Rockleigh high (isolate BG)	41.007620, −73.940000	SRR22223271	JAPFCF000000000	6,049,096	111	61.84	1,923,978	5,601	5,518	70	MF111944.1	Pseudomonas putida (CP022561.1)
Moturis untreated (isolate BI)	41.015904, −73.937346	SRR22223270	JAPFCG00000000	5,947,371	101	62.11	1,655,760	5,490	5,405	70	KC013979.1	Pseudomonas putida (CP022561.1)
Moturis high (isolate BZ)	41.015904, −73.937346	SRR22223269	JAPFCH00000000	6,296,982	189	61.55	1,429,439	5,937	5,853	70	MF111944.1	Pseudomonas putida (CP043576.1)
Moturis high (isolate CB)	41.015904, −73.937346	SRR22223268	JAPFCI000000000	6,285,243	152	61.56	1,981,765	5,915	5,833	70	MT102287.1	Pseudomonas putida (CP043576.1)
Marsh untreated (isolate E)	41.038606, −73.915210	SRR22223267	JAPFCJ000000000	6,004,388	164	61.83	1,797,532	5,548	5,456	70	MF111944.1	Unclassified Pseudomonas (CP044546.1)
Marsh high (isolate Q)	41.038606, −73.915210	SRR22223265	JAPFCL000000000	6,007,150	228	61.81	1,395,356	5,569	5,473	70	MF111944.1	Unclassified Pseudomonas (CP044546.1)
Pseudomonas putida (*c*ontrol)	NA^*a*^	SRR22223266	JAPFCK000000000	6,133,470	164	62.37	1,642,352	5,621	5,522	69	NA	Pseudomonas putida (AP013070.1)

a NA, not applicable.

Cells were grown in Luria broth at 32°C for 24 h prior to sedimentation. Genomic DNA was extracted from cell pellets using the Qiagen QIAmp DNA kit and high-throughput (HT) DNA kit. DNA was quantified using the Qubit 2.0 fluorometer, and integrity was verified on a 1% agarose gel. The NEBNext Ultra DNA library preparation kit for Illumina, clustering, and sequencing reagents were used following the manufacturer’s recommendations. DNA was fragmented by acoustic shearing, end repaired, and adenylated. Adapters were ligated, followed by enrichment using a limited-cycle PCR. The library was validated using D1000 ScreenTape and quantified using real-time PCR and a Qubit 2.0 fluorometer. Samples were sequenced on an Illumina MiSeq instrument at Azenta Life Sciences, using a 2 × 150-bp paired-end configuration. Image analysis and base calling were conducted by the MiSeq Control Software (8). Raw sequencing data (bcl files) were converted into fastq files and demultiplexed using Illumina bcl2fastq software. Reads were assessed for quality using FastQC v0.11.5 (9). Low-quality reads and adapter regions were filtered using the bcl2fastq tool v2.20.0.

*De novo* genome assembly was performed through the Bacterial and Viral Bioinformatics Resource Center (BV-BRC) v3.6.2 (https://www.bv-brc.org). The assembly was performed using SPAdes v3.13.0 (10) with two Pilon iterations (11). Assembled contigs were then run through the NCBI contamination screen, which found sequences that were trimmed and/or excluded. Contigs were annotated using the NCBI Prokaryotic Genome Annotation Pipeline (PGAP) v6.3 (12). Default software parameters were used unless otherwise specified. Assembly and annotation statistics for contigs of >300 bp are presented in Table 1. Species identity was determined by performing a BLASTn search, using the largest contig, against NCBI taxonomic identifier 136845 (Pseudomonas). These strains add to the genomic data available for the Pseudomonas genus, supporting further investigation into their use as indicators of glyphosate contaminations of soil and water.

### Data availability.

The assembled sequences and annotations were deposited in GenBank. Raw reads and assembled genomes are available under BioProject accession number PRJNA898300.
